# Vps34-mediated macropinocytosis in Tuberous Sclerosis Complex 2-deficient cells supports tumorigenesis

**DOI:** 10.1038/s41598-018-32256-x

**Published:** 2018-09-21

**Authors:** Harilaos Filippakis, Amine Belaid, Brian Siroky, Constance Wu, Nicola Alesi, Thomas Hougard, Julie Nijmeh, Hilaire C. Lam, Elizabeth P. Henske

**Affiliations:** 1 Pulmonary and Critical Care Medicine, Department of Medicine, Brigham and Women’s Hospital and Harvard Medical School, Boston, MA USA; 2 Division of Nephrology and Hypertension, Cincinnati Children’s Hospital Medical Center, Cincinnati, OH USA

## Abstract

Tuberous Sclerosis Complex (TSC), a rare genetic disorder with mechanistic target of rapamycin complex 1 (mTORC1) hyperactivation, is characterized by multi-organ hamartomatous benign tumors including brain, skin, kidney, and lung (Lymphangioleiomyomatosis). mTORC1 hyperactivation drives metabolic reprogramming including glucose and glutamine utilization, protein, nucleic acid and lipid synthesis. To investigate the mechanisms of exogenous nutrients uptake in Tsc2-deficient cells, we measured dextran uptake, a polysaccharide internalized via macropinocytosis. Tsc2-deficient cells showed a striking increase in dextran uptake (3-fold, p < 0.0001) relative to Tsc2-expressing cells, which was decreased (3-fold, p < 0.0001) with mTOR inhibitor, Torin1. Pharmacologic and genetic inhibition of the lipid kinase Vps34 markedly abrogated uptake of Dextran in Tsc2-deficient cells. Macropinocytosis was further increased in Tsc2-deficient cells that lack autophagic mechanisms, suggesting that autophagy inhibition leads to dependence on exogenous nutrient uptake in Tsc2-deficient cells. Treatment with a macropinocytosis inhibitor, ethylisopropylamiloride (EIPA), resulted in selective growth inhibition of Atg5-deficient, Tsc2-deficient cells (50%, p < 0.0001). Genetic inhibition of autophagy (Atg5^−/−^ MEFs) sensitized cells with Tsc2 downregulation to the Vps34 inhibitor, SAR405, resulting in growth inhibition (75%, p < 0.0001). Finally, genetic downregulation of Vps34 inhibited tumor growth and increased tumor latency in an *in vivo* xenograft model of TSC. Our findings show that macropinocytosis is upregulated with Tsc2-deficiency via a Vps34-dependent mechanism to support their anabolic state. The dependence of Tsc2-deficient cells on exogenous nutrients may provide novel approaches for the treatment of TSC.

## Introduction

Tuberous Sclerosis Complex (TSC) is a multisystem hamartomatous disease in which tumors develop in multiple organs, including the brain (subependymal giant cell astrocytomas), heart (rhabdomyomas), kidney (angiomyolipomas and renal cell carcinoma), skin (angiofibromas) and lung (lymphangioleiomyomatosis)^1,2^. TSC is caused by germline mutations in either the *TSC1* or *TSC2* genes^3,4^. The TSC1 and TSC2 proteins (hamartin and tuberin, respectively) function together with TBC1D7 to regulate the mammalian target of rapamycin complex 1 (mTORC1)^5^. mTORC1 is a master regulator of cellular metabolic homeostasis, integrating nutrient signals to stimulate anabolic metabolism. Activation of mTORC1 is stimulated by signals that include growth factors and intracellular amino acids resulting in its translocation to lysosomal membranes^6–8^. mTORC1 phosphorylates multiple downstream targets to enhance ribosome biogenesis and protein translation, increase nucleotide and lipid synthesis, and inhibit autophagy initiation^9,10^.

In TSC, constitutive activation of mTORC1 leads to extensive metabolic reprogramming and vulnerabilities that can create therapeutic opportunities. For example, rewiring of guanylate nucleotide synthesis in Tsc2-deficient cells leads to sensitivity to mizoribine, an Inosine-5′-monophosphate dehydrogenase (IMPDH) inhibitor, while inhibition of mTORC1-dependent autophagy in Tsc2-deficient cells leads to specific sensitivity to further autophagy/lysosomal inhibition and to inhibition of the pentose phosphate pathway^11,12^.

In parallel with these data on the impact of intracellular metabolic reprogramming on TSC2-deficient cells, the uptake and utilization of extracellular nutrients is also altered by mTORC1 hyperactivity. mTORC1-hyperactive cells have been shown to be highly dependent on the uptake of extracellular glucose and glutamine, leading to sensitivity to inhibitors of glycolysis and glutaminolysis^13,14^. We have recently found that treatment of Tsc2-deficient cells with the lysosomal inhibitor chloroquine enhances the mevalonate pathway and the uptake of exogenous lipoproteins, creating a scenario in which Tsc2-deficient cells are resistant to treatment with statins^15^.

Extracellular proteins can provide fuel for the high anabolic demands of proliferating cells via macropinocytosis^16,17^, a conserved, actin-dependent endocytic process by which cells incorporate extracellular fluids into large vesicles called macropinosomes^16,18,19^. Macropinocytosis can be stimulated by receptor tyrosine kinases including epidermal growth factor receptor (EGFR), and by oncogenes including Ras and v-Src^20,21^. Macropinocytosis is distinguished from other forms of endocytosis by its susceptibility to inhibitors of Na^+^/H^+^ exchange^22^. In tumor cells with oncogenic Ras mutations, macropinocytosis allows the internalization of extracellular protein, which is delivered to the lysosome for degradation^16,17^. Macropinocytic activity is increased in mouse models of K-Ras driven pancreatic ductal adenocarcinomas^23^. Endocytic trafficking via macropinocytosis is a clathrin-independent process that results in delivery of macropinocytic cargo to the lysosome for degradation. Vps34 is a PI3KC3 lipid kinase and is the only class III PI-3-kinase in mammals responsible for producing phosphatidylinositol-3-phosphate (PI(3)P), thereby mediating endocytic trafficking of nutrients to the lysosome^24^. The role of Vps34 in macropinocytic uptake of nutrients is poorly understood in mTORC1-hyperactive tumors.

Here, we investigated the impact of hyperactive mTORC1 on macropinocytosis in Tsc2-deficient cells. We found that macropinocytosis is enhanced in Tsc2-deficient cells, in contrast to the mTORC1-dependent inhibition of macropinocytosis observed in K-Ras mutant cells. Importantly, the enhanced macropinocytosis in Tsc2-deficient cells promotes cell proliferation via a Vps34-dependent-mechanism, leading to potential therapeutic opportunities for TSC-associated tumors.

## Results

### Macropinocytosis is increased in Tsc2-deficient cells

**Figure 1:**
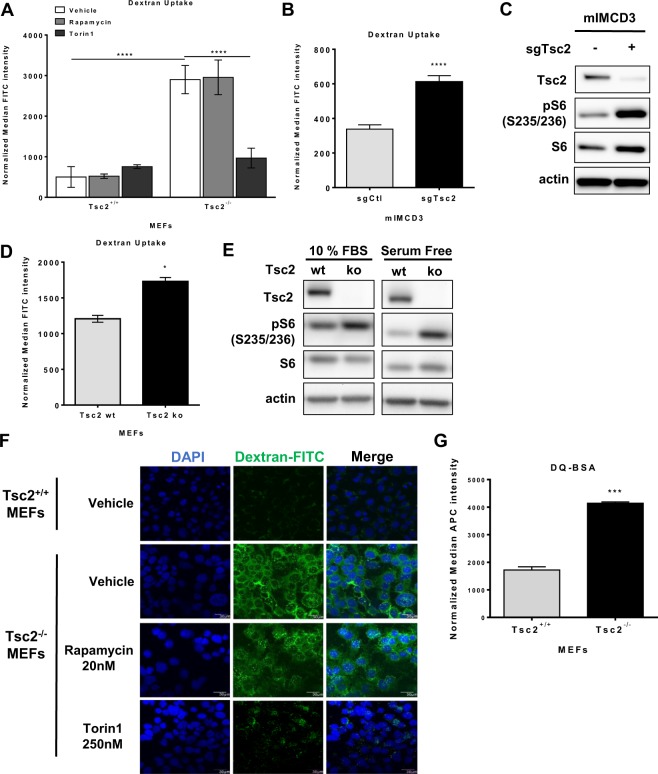
Macropinocytosis is increased in Tsc2-deficient cells. (**A**) Uptake of macropinocytotic cargo dextran (FITC-Dextran, 70 kDa; 0.5 mg/ml) was significantly increased in Tsc2^−/−^ MEFs compared to Tsc2^+/+^ MEFs. Rapamycin (20 nM) had no impact on macropinocytosis in Tsc2^−/−^ MEFs, while Torin1 (250 nM) reduced dextran uptake to levels comparable to Tsc2^+/+^ MEFs. (**B**) Increased dextran uptake in mouse inner medullary collecting duct epithelial cells (mIMCD3) with Tsc2 knockout (sgTsc2) compared to sgCtl cells. (**C**) Immunoblot of serum starved mIMCD3 cells showing knockout of Tsc2 and increased phosphorylation of S6 ribosomal protein. The blot was cropped to highlight the relevant bands. The full-length blot is presented in Supplementary Figure 2B. (**D**) Increased dextran uptake in Tsc2-deficient embryonic fibroblasts (MEFs) derived from Tsc2^fl/fl^ Rosa26-CreERT2 mice (Tsc2ko) compared to Tsc2-expressing MEFs (Tsc2wt). (**E**) Immunoblot of MEFs treated with ethanol or 4-hydroxytamoxifen to knockout Tsc2. Cells were either grown in 10% FBS DMEM or serum starved for 16 hours. (**F**) Representative images of Tsc2^+/+^ and Tsc2^−/−^ MEFs treated for 24 hours with vehicle, rapamycin (20 nM) or Torin1 (250 nM). The macropinocytic cargo dextran was internalized at higher levels in Tsc2^−/−^ MEFs, compared to Tsc2^+/+^ MEFs. Rapamycin did not impact macropinocytosis in Tsc2^−/−^ MEFs. FITC-Dextran uptake decreased with Torin1. (**G**) Increased DQ Red BSA processing in Tsc2^−/−^ MEFs, compared to Tsc2^+/+^ MEFs. Data represented as mean +/− standard deviation of three biological replicates. Statistical significance was assessed using two-way and one-way ANOVAs with Bonferroni correction with *p < 0.05, ***p < 0.001, ****p < 0.0001.

To examine whether macropinocytosis is regulated by Tsc2, Tsc2^+/+^ and Tsc2^−/−^ MEFs (originally derived from littermate control animals^25^) were treated with rapamycin (20 nM) to inhibit mTORC1, Torin1 (250 nM) to inhibit mTORC1 and mTORC2, or vehicle (DMSO) for 24 hours. Cells were then incubated with 70 kDa fluorescein isothiocyanate-conjugated dextran (FITC-dextran; 0.5 mg/ml) for one hour. Macropinocytic uptake, measured by median FITC-dextran positive cells using flow cytometry, was increased by 3-fold (p < 0.0001) in Tsc2^−/−^ MEFs compared to wildtype cells (Fig. 1A). Interestingly, in Tsc2^−/−^ MEFs, Torin1 reduced uptake by 3.2-fold (p < 0.0001), to levels comparable to wildtype cells. Rapamycin treatment did not affect dextran uptake in Tsc2-deficient cells and neither rapamycin nor Torin1 affected dextran uptake in Tsc2-expressing cells. We next tested the impact of rapamycin or Torin1 on dextran uptake following acute treatments. Macropinocytic uptake was unchanged in Tsc2^−/−^ or Tsc2^+/+^ MEFs treated with rapamycin or Torin1 for 1 hour, despite being sufficient to inhibit mTOR signaling at this time point (Supplementary Fig. 1A,B). To confirm that the increased macropinocytosis phenotype in the Tsc2-deficient MEFs is caused by loss of Tsc2, we performed dextran uptake assays on two additional models of Tsc2 deficiency with Tsc2-expressing controls: mouse inner medullary collecting duct 3 epithelial cells (mIMCD3) cells (Fig. 1B) and MEFs with tamoxifen-inducible loss of Tsc2 (Fig. 1D). Macropinocytosis was elevated in both cell lines with Tsc2-deficiency. Dextran uptake was increased by 1.7-fold (p < 0.0001) in mouse inner medullary collecting duct epithelial cells (mIMCD3) with Tsc2 knockout (sgTsc2) compared to sgCtl cells (Fig. 1B,C). Additionally, dextran uptake was increased (1.4-fold, p < 0.05) in Tsc2-deficient MEFs (Tsc2ko) derived from Tsc2^fl/fl^ Rosa26-CreERT2 mice compared to Tsc2-expressing MEFs (Tsc2wt), (Fig. 1D,E). The relative dextran uptake was different among the three models of Tsc2-deficiency, possibly due to differences in each cell type. However, macropinocytosis was increased in all cells with Tsc2-deficiency.

We next assessed dextran internalization by confocal fluorescence microscopy. Tsc2^+/+^ and Tsc2^−/−^ MEFs were incubated with 70 kDa FITC-dextran (0.5 mg/ml) for 30 minutes. Macropinosomes identified as FITC-dextran positive puncta were markedly increased in Tsc2^−/−^ MEFs compared to Tsc2^+/+^ MEFs (Fig. 1F). Dextran uptake was reduced in Tsc2^−/−^ MEFs treated with Torin1 (250 nM) for 24 hours, while rapamycin (20 nM) had no impact on macropinosome puncta in Tsc2^−/−^ MEFs, confirming our flow cytometry data.

Nutrient scavenging via macropinocytosis is a crucial mechanism of nutrient acquisition that supports proliferation of tumors, especially in nutrient-poor environments. To determine whether exogenous protein uptake through macropinocytosis can be processed by Tsc2-deficient cells, Tsc2^+/+^ and Tsc2^−/−^ MEFs were grown in DMEM supplemented with 1% FBS and then incubated for 1 hour with a BODIPY fluorescently-labelled form of bovine serum albumin (DQ Red BSA; 0.2 mg/ml). DQ Red BSA is a self-quenching fluorescent dye that emits fluorescence following proteolytic cleavage at the lysosome. BSA fluorescent intensity was increased 2.4-fold in Tsc2-deficient cells (p < 0.001) compared to Tsc2-expressing cells, indicating that Tsc2-deficient cells can scavenge and process exogenous protein via the lysosome (Fig. 1G). These data suggest that Tsc2-deficient cells are characterized by increased lysosomal degradation and macropinocytosis.

### Macropinocytosis is enhanced by autophagy inhibition selectively in Tsc2^−/−^ MEFs

**Figure 2:**
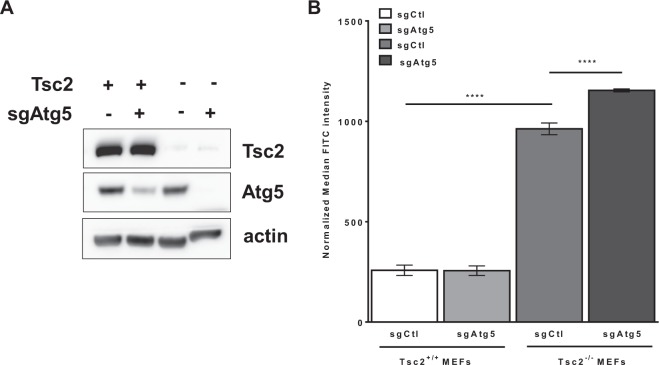
Macropinocytosis is further increased by autophagy inhibition in Tsc2-deficient cells. (**A**) Immunoblots confirmed Atg5 knockout in Tsc2^+/+^ and Tsc2^−/−^ MEFs. The blot was cropped to highlight the relevant bands. The full-length blot is presented in Supplementary Fig. 3. (**B**) Macropinocytosis was increased in Tsc2^−/−^ sgAtg5 MEFs compared to Tsc2^−/−^ sgCtl MEFs. Median fluorescent intensity of FITC-Dextran was quantified by flow cytometry. Data represented as mean +/− standard deviation of three biological replicates. Statistical significance was assessed using two-way and one-way ANOVAs with Bonferroni correction with ****p < 0.0001.

Previously, we have found that metabolic reprogramming of Tsc2-deficient cells is autophagy-dependent^12^. To determine whether autophagy is involved in the macropinocytic uptake of nutrients in TSC, we generated Tsc2^+/+^ and Tsc2^−/−^ MEFs with Atg5 knockout, using CRIPSR/Cas9. Loss of Atg5 was confirmed by immunoblot using an Atg5 antibody that recognizes the Atg5-Atg12 conjugated protein (Fig. 2A). Macropinocytosis was assessed by dextran uptake at 60 minutes in the four cell lines: Tsc2^+/+^ sgCtl, Tsc2^+/+^ sgAtg5, Tsc2^−/−^ sgCtl and Tsc2^−/−^ sgAtg5 MEFs. As observed previously, dextran uptake was ~4-fold higher in the Tsc2^−/−^ sgCtl MEFs compared with Tsc2^+/+^ sgCtl MEFs (Fig. 2B). Knockout of Atg5 in Tsc2^−/−^ MEFs further increased dextran uptake by 22% (p < 0.0001), compared to Tsc2^−/−^ sgCtl MEFs. Importantly, Atg5 deletion in Tsc2^+/+^ MEFs did not affect dextran uptake (Fig. 2B). In summary, these data suggest that autophagy inhibition selectively enhances macropinocytosis in Tsc2-deficient cells, reflecting the high anabolic demands associated with mTORC1 hyperactivation.

### Macropinocytic uptake is mediated via Vps34 in Tsc2-deficient cells

**Figure 3:**
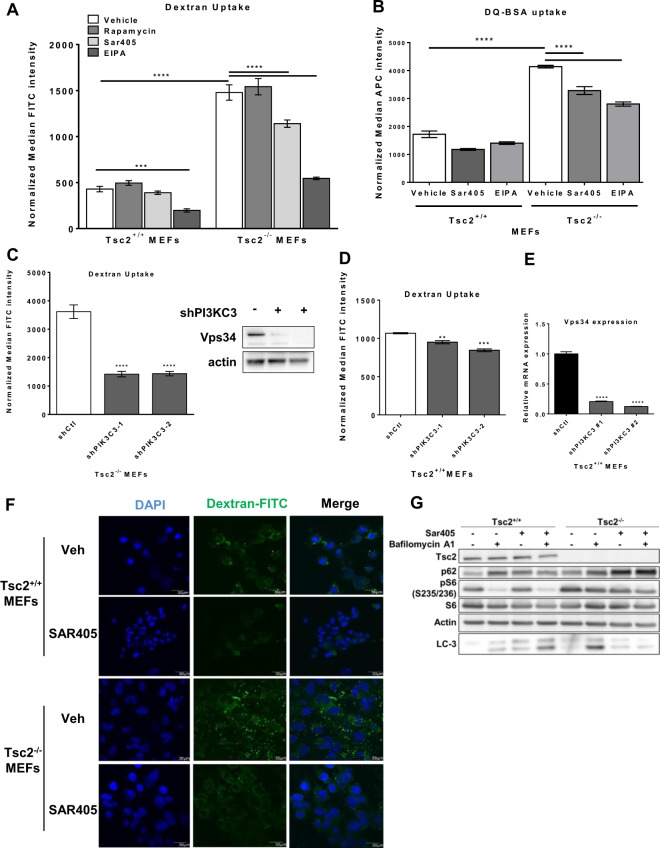
Enhanced macropinocytosis in Tsc2-deficient cells is Vps34-dependent. (**A**) Vps34 inhibitor SAR405 (2 uM, 24 hours) and the macropinocytosis inhibitor EIPA (25 uM, 24 hours) decreased macropinocytosis in Tsc2^−/−^ MEFs. Rapamycin (20 nM, 24 hours) had no effect on dextran uptake. (**B**) Uptake and lysosomal processing of exogenous albumin is enhanced in Tsc2-deficient cells. SAR405 (2 uM, 24 hours) and EIPA (25 uM, 24 hours) decreased uptake of macropinocytic cargo DQ-BSA. (**C**) Vps34 downregulation in Tsc2^−/−^ MEFs reduced uptake of dextran. The blot was cropped to highlight the relevant bands. The full-length blot is presented in Supplementary Figure 4. (**D**) Vps34 downregulation in Tsc2^+/+^ MEFs had no effect on dextran uptake. (**E**) Quantitative real-time PCR validation of Vps34 knockdown. (**F**) Confocal microscopy revealed reduced FITC-Dextran positive macropinosomes in Tsc2^−/−^ MEFs treated with SAR405 (2 uM, 24 hours). (**G**) Autophagic flux was decreased in Tsc2^−/−^ MEFs following SAR405 treatment (2 uM, 24 hours), while autophagy was mildly increased in Tsc2^+/+^ MEFs. Cells were treated with bafilomycin A1 (50 nM) for 5 hours prior to harvesting. The blot was cropped to highlight the relevant bands. The full-length blot is presented in Supplementary Fig. 4. Data represented as mean +/− standard deviation of three biological replicates. Statistical significance was assessed using two-way and one-way ANOVAs with Bonferroni correction with ***p < 0.001, ****p < 0.0001.

Vps34 (PI3KC3) is a lipid kinase of the PI3K family that is required for endosomal trafficking and autophagy, thereby regulating delivery of endocytic and autophagic cargo to the lysosome^26,27^. Previously, we found that the combination of Vps34 inhibition (using SAR405) and lysosomal inhibition (using chloroquine) blocked cholesterol uptake in Tsc2-deficient cells, but not Tsc2-expressing cells, suggesting a selective role of Vps34-dependent trafficking of exogenous nutrients in the Tsc2-deficient setting^15^. To determine whether Vps34 is involved in the enhanced macropinocytic nutrient uptake of Tsc2-deficient cells, we treated cells with SAR405 (2 uM; 24 hours) and quantified dextran uptake by flow cytometry^28^. Dextran uptake was 3.8-fold higher (p < 0.0001) in untreated Tsc2-deficient cells compared to Tsc2-expressing cells, as expected (Fig. 3A). SAR405 treatment inhibited dextran uptake (25%, p < 0.0001) in Tsc2-deficient cells, with no effect on the Tsc2-expressing cells (Fig. 3A). In agreement with our previous observations (Fig. 1A), rapamycin did not affect uptake of dextran. Treatment with a macropinocytosis inhibitor EIPA (25 uM; 24 hours) decreased dextran uptake by ~60%, close to the level of the Tsc2-expressing cells (Fig. 3A). To investigate the role of Vps34 and macropinocytosis on the uptake and lysosomal processing of exogenous protein, we measured protein uptake using DQ-BSA. Uptake of exogenous albumin was increased 2.5-fold (p < 0.0001) in Tsc2-deficient cells compared to Tsc2-expressing cells and was repressed following Vps34 (20%, p < 0.0001) or EIPA treatment (30%, p < 0.0001; Fig. 3B). To confirm that the decrease in macropinocytosis is Vps34-dependent, we generated Tsc2^+/+^ and Tsc2^−/−^ MEFs with stable downregulation of Vps34 using two shRNA clones (shPI3KC3-1 and shPI3KC3-2). Macropinocytosis was reduced approximately 2.5-fold (p < 0.0001) in both Vps34 shRNA clones compared to Tsc2^−/−^ shCTL MEFs, while downregulation of Vps34 in Tsc2^+/+^ MEFs had no effect on dextran uptake (Fig. 3C,D). Using confocal microscopy, we observed a striking decrease of dextran uptake in Tsc2-deficient cells upon Vps34 inhibition using SAR405 (2 uM; 24 hours), while macropinosome puncta were unchanged in Tsc2-expressing MEFs (Fig. 3F). These data suggest that the enhanced macropinocytosis in Tsc2-deficient cells is partially mediated by Vps34.

To assess the impact of Vps34 inhibition on autophagy, we performed an autophagic flux assay. We found that Vps34 inhibition with SAR405 inhibits autophagic flux in Tsc2-deficient cells, as seen by LC3-II levels and accumulation of p62 (Fig. 3G). In contrast, Vps34 inhibition mildly increases autophagic flux in Tsc2-expressing cells. In summary, these data support a role for Vps34 in the regulation of both autophagy and macropinocytosis selectively in Tsc2-deficient cells.

### Autophagy inhibition synergizes with Vps34 inhibition or macropinocytosis inhibition to selectively inhibit the proliferation of Tsc2-deficient cells

**Figure 4:**
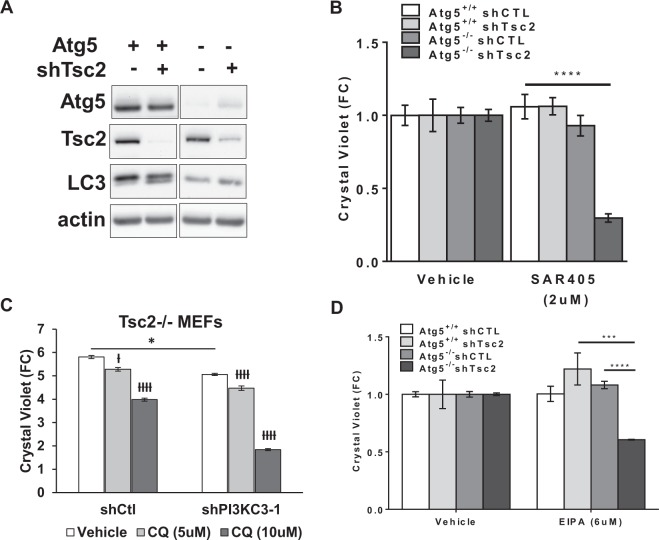
Autophagy inhibition synergizes with Vps34 inhibition or macropinocytosis inhibition to selectively inhibit proliferation of Tsc2-deficient cells. (**A**) Immunoblots confirmed Tsc2 downregulation in Atg5^+/+^ and Atg5^−/−^ MEFs. The blot was cropped to highlight the relevant bands. The full-length blot is presented in Supplementary Figure 5A. (**B**) Proliferation of Atg5^−/−^ shTsc2 MEFs grown in 1% FBS DMEM was selectively inhibited by Vps34 inhibitor SAR405 (2 uM, 48 hours). (**C**) Vps34 downregulation sensitized Tsc2^−/−^ MEFs to CQ treatment (5 uM, 72 hours). (**D**) Proliferation of Atg5^−/−^ shTsc2 MEFs grown in 1% FBS DMEM was selectively inhibited by the macropinocytosis inhibitor EIPA (6 uM, 48 hours). Data represented as mean +/− standard deviation of six biological replicates. Statistical significance was assessed using two-way and one-way ANOVAs with Bonferroni correction with *p < 0.05, ***p < 0.001, ****p < 0.0001. ^ƚ^p < 0.05, ^ƚƚƚƚ^p < 0.0001 when comparing treated to vehicle treatment in same cell type.

Atg5 deletion inhibited autophagy and enhanced macropinocytosis (Fig. 2B), while Vps34 inhibition decreased both autophagy and macropinocytosis in Tsc2-deficient cells (Fig. 3A–C). Therefore, we asked whether complete autophagy inhibition by Atg5 knockout would sensitize Tsc2-deficient cells to Vps34 inhibition by SAR405. Atg5^+/+^ and Atg5^−/−^ MEFs with shRNA downregulation of Tsc2 or control shRNA (Fig. 4A) were treated with SAR405 for 48 hours. SAR405 treatment reduced the proliferation of the Atg5^−/−^ shTSC2 cells by 75% (p < 0.0001; Fig. 4B). In contrast, SAR405 had no effect on the proliferation of Atg5^+/+^ cells (regardless of TSC2 expression status). This finding suggests that autophagy inhibition creates a dependency on exogenous nutrients and sensitizes Tsc2-deficient cells to Vps34 inhibition by SAR405. To determine the role of lysosomal degradation in the proliferation of Tsc2^−/−^ shPIK3C3 MEFs we treated with the lysosomal/autophagy inhibitor CQ. Interestingly, treatment with CQ (5 uM and 10 uM; 72 hours) further decreased the proliferation of Tsc2^−/−^ shPIK3C3 MEFs (2.5-fold, p < 0.0001, Fig. 4C). In summary, these data suggest that autophagy, macropinocytosis and lysosomal activity ensure the viability of Tsc2-deficient cells.

We next investigated whether the macropinocytosis inhibitor EIPA could inhibit the proliferation of Tsc2-deficient cells. EIPA treatment (6 uM; 48 hours) reduced proliferation of Atg5^−/−^ shTsc2 MEFs by ~50%, without affecting autophagy proficient or Tsc2-expressing cells (Fig. 4D). In sum, these data suggest that Vps34 plays a crucial role in supporting the survival of Tsc2-deficient cells, especially under conditions of autophagy inhibition. Taken together, these data suggest that nutrient uptake in Tsc2-deficient cells is Vps34-dependent, particularly under conditions of metabolic stress such as autophagy-deficiency.

### Vps34 downregulation suppresses Tsc2-deficient tumor growth

**Figure 5:**
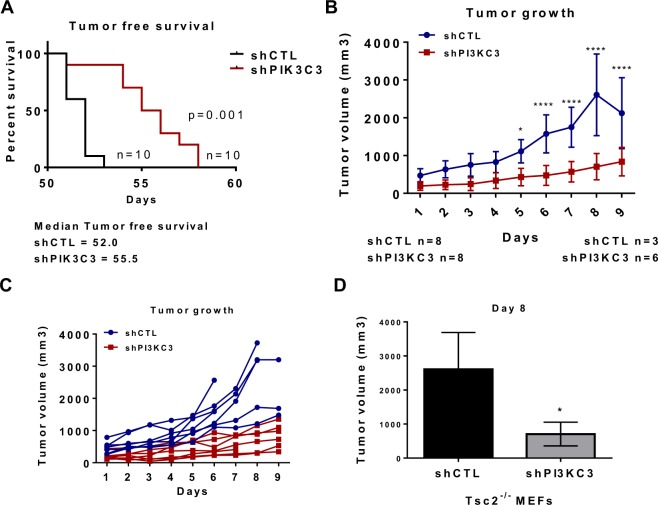
Vps34 downregulation decreases proliferation of Tsc2-deficient cells *in vivo*. Mice were injected subcutaneously with 2.5 × 10^6^ Tsc2^−/−^ shCTL or shPI3KC3 MEFs. Mice bearing tumors > 100 mm^3^ were identified by palpation. (**A**) Tumor latency was increased in Tsc2^−/−^ shPIK3C3, compared to Tsc2^−/−^ shCTL tumors. Statistical significance was assessed by Mantel-Cox Test with p = 0.001. (**B**) Tumor volume was decreased in the shPI3KC3 tumors compared to shCTL tumors. (**C**) Growth of individual shCTL and shPI3KC3 tumors. (**D**) Tumor volume at 8 days after palpation. Data presented as mean +/− standard deviation. Statistical significance was assessed by Student’s unpaired t-test with *p < 0.05, ****p < 0.0001.

To determine whether Tsc2-deficient cells are Vps34-dependent *in vivo*, we inoculated 2.5 × 10^6^ Tsc2-deficient cells with downregulation of Vps34 (shPIK3C3) or shCTL subcutaneously into NOD/SCID/IL2gR deficient mice (n =10  mice/group) and monitored tumor growth. Vps34 downregulation significantly increased tumor-free survival, (p < 0.001, Fig. 5A). Inhibition of Vps34 repressed tumor growth (Fig. 5B,C) and the shPI3KC3 tumors were approximately one third the size of shCTL tumors at day 8 after initial palpation (Fig. 5D). These data suggest that Vps34 supports tumorigenic growth of Tsc2-deficient cells *in vivo* and provide evidence that targeting Vps34 may have therapeutic potential in mTORC1-driven tumors.

## Discussion

Tuberous sclerosis complex is a multisystem disease characterized by constitutive activation of mTORC1. mTORC1 is a central signaling hub, incorporating nutrient signals from the environment to regulate cellular growth and metabolism^7^. Tsc2-deficient cells have been previously found to have altered uptake of specific extracellular nutrients, including glucose, glutamine, and lipids. The bulk uptake of extracellular protein and nutrients via macropinocytosis is known to be mTORC1-dependent in K-Ras driven tumors, resulting in a striking and unexpected enhancement of tumor growth upon mTORC1 inhibition^16,17,23,29,30^. Whether macropinocytosis is mTORC1-dependent in Tsc2-deficient cells and tumors has not been previously investigated. Here, we show that macropinocytosis and exogenous protein uptake are enhanced in Tsc2-deficient cells, in contrast to the findings in the context of oncogenic K-Ras. In TSC, macropinocytic uptake supports the survival of mTORC1-hyperactive cells via a Vps34-dependent mechanism, *in vitro* and *in vivo*. These findings may have clinical implications for the therapy of TSC-associated tumors. For example, targeting multiple nutrient uptake pathways with endosomal and lysosomal inhibitors has proven to be a successful therapeutic strategy in *Ras* and PTEN-driven tumor cells^31,32^.

Using flow cytometry and confocal imaging approaches, we found that macropinocytosis and lysosomal processing of exogenous albumin are increased in Tsc2-deficient cells (Fig. 1). Interestingly, inhibition of mTORC1 with rapamycin did not impact macropinocytosis in Tsc2-deficient cells, while treatment with Torin1, an ATP-competitive inhibitor of mTOR, reduced macropinocytic uptake to levels comparable with Tsc2-expressing cells. It is surprising that macropinocytosis in Tsc2-deficient cells is inhibited by Torin1, but not by Rapamycin. This suggests an mTORC2-dependent mechanism, although further studies would be required to prove this hypothesis. The short and long-term impact of mTORC1, mTORC2 or SAR405 inhibition on macropinocytosis are summarized in Supplementary Table 1. These findings appear to be in agreement with recent studies of *K-ras*^*G12D*^ MEFs in which mTORC1 activation drives extracellular protein uptake via macropinocytosis^23^. Previous studies have shown that under conditions of nutrient starvation, inactivation of mTORC1 alleviates repression of the autophagy initiation kinases Ulk1/2^9,10^. This in turn enhances autophagic degradation of nutrients at the lysosome and promotes cell proliferation. In Tsc2-deficient cells, constitutive mTORC1 activation results in continued suppression of autophagy^11^. Therefore, our findings suggest that in Tsc2 deficiency the low turnover of intracellular nutrients acquired via autophagy creates metabolic stress which is compensated by uptake of exogenous nutrients via macropinocytosis.

Previously, we and others have found that autophagy plays a key role in the maintenance of metabolic homeostasis in Tsc2-deficient cells and in their survival, both *in vitro* and *in vivo* models^11,33^. In *Ras*-transformed cells, deletion of Ulk1/2 does not affect macropinocytosis^17^, but we found that downregulation of Atg5 further enhances macropinocytosis in Tsc2-deficient cells by ~20% (Fig. 2). Interestingly, while Atg5 impacts dextran uptake in Tsc2-deficient cells, no effect was seen in Tsc2-expressing cells. This may reflect the extensive metabolic reprogramming that occurs in Tsc2-deficient cells, in part to compensate for the constitutively low autophagy. These findings further distinguish the regulation of macropinocytosis in Ras-transformed vs. Tsc2-deficient cells and support the role of macropinocytosis as an alternative nutrient acquisition pathway.

We next hypothesized that Vps34 sustains viability of Tsc2-deficient cells by mediating macropinocytic uptake of nutrients to the lysosome. Vps34 forms distinct kinase complexes (PI3KC3-C1 and PI3KC3-C2) that play important roles in both endolysosomal sorting and initiation of autophagy, respectively^27,34^. Using both pharmacologic and genetic approaches, we found that macropinocytosis is Vps34-dependent in Tsc2-deficient but not in Tsc2-expressing cells (Fig. 3). Genetic inhibition of Vps34 did not completely abolish macropinocytosis in Tsc2-deficient cells. Therefore, it is possible that other mechanisms of nutrient uptake complement the Vps34-dependent mechanism described here. In Tsc2-deficient cells, Vps34 inhibition decreases autophagy and macropinocytosis, while Atg5 deletion increases macropinocytosis. Therefore, we hypothesize that the impact of Vps34 on macropinocytosis over-rides its effects on autophagy in Tsc2-deficient cells. Interestingly, Atg5 downregulation selectively sensitized Tsc2-deficient cells to the Vps34 inhibitor SAR405, suggesting that endocytic trafficking plays a crucial role in the viability of Tsc2-deficient cells when autophagy is abrogated, and perhaps also in settings of metabolic stress (Fig. 4B and C). In pancreatic cancer cells, deprivation of extracellular protein uptake by macropinocytosis inhibition represents a potential therapeutic approach, creating a shortage of amino acids^29^. This concept is consistent with our finding that inhibition of macropinocytosis using EIPA selectively inhibited the proliferation of Atg5^−/−^ shTsc2 MEFs (Fig. 4D). These observations suggest that macropinocytosis and endocytic trafficking via Vps34 are potential therapeutic targets in tuberous sclerosis complex. To address this, we inoculated Tsc2^−/−^ MEFs with downregulation of Vps34 into mice. Tsc2^−/−^ shPI3KC3 xenograft tumors had delayed onset and markedly decreased growth compared to Tsc2^−/−^ shCtl tumors, indicating that Vps34 plays an important role in Tsc2-driven tumors (Fig. 5A–D).

mTORC1 inhibitors are effective cytostatic agents for the treatment of mTORC1-hyperactive tumors, including tumors in patients with TSC. However, tumors regrow upon cessation, necessitating lifelong treatment^35,36^. This generates an unmet need for novel therapeutic approaches that exert a cytocidal response in mTORC1-hyperactive tumors. Tsc2-deficient cells are characterized by low levels of autophagy, which limit the degradation of intracellular macromolecules and turnover of nutrients. Our data suggest that upregulation of macropinocytosis provides exogenous nutrients to compensate for low levels of autophagy. Taken together, our findings suggest that simultaneous targeting of nutrient access pathways such as autophagy and macropinocytosis may provide a therapeutic avenue for the treatment of TSC.

## Materials and Methods

### Cell lines and culture conditions

Tsc2^−/−^p53^−/−^ and Tsc2^+/+^p53^−/−^ mouse embryonic fibroblasts (MEF) were provided by David Kwiatkowski (Brigham and Women’s Hospital, Boston, MA). MEFs were isolated from Tsc2^flox/flox^-Rosa26-CreERT2 embryos and clones with knockout of *Tsc2* (Tsc2 KO MEFs) as described previously^37^. Cells treated with ethanol were used as controls (Tsc2 WT MEFs). Tsc2^−/−^p53^−/−^ and Tsc2^+/+^p53^−/−^ MEFs were infected with lentivirus (pLX_311Cas9v2) to stably express CRISPR/Cas9 (Broad Institute, Cambridge, MA, USA). Mouse embryonic fibroblasts were transfected with a plasmid containing guide RNA sequences against mouse Atg5 or scramble guide RNAs and selected using blasticidin (10ug/ml) as described previously^38^. Single cell clones were sorted into separate wells for expansion (Dana-Farber Cancer Institute Flow Cytometry Core). Immunoblotting was used to confirm the loss of Atg5. Mouse inner medullary epithelial cells (mIMCD-3) were transfected with a CRISPR plasmid with green fluorescent protein (GFP) and guide RNA sequences targeting Tsc2, constructed by the Cincinnati Children’s Hospital Medical Center (CCHMC) Transgenic Animal Genome Editing Core Facility and selected using flow cytometry for GFP-positive clones. Polymerase chain reaction was used to amplify the targeted region and validate knockout of both Tsc2 alleles. All cells tested negative for mycoplasma contamination using MycoAlert (Lonza, Walkersville, MD) and were re-tested monthly. For immunoblot experiments cells were grown in serum-free (no FBS) conditions for 16 hours. Cells were cultured at 37 °C in 5% CO_2_ in DMEM supplemented with 10% FBS and gentamycin sulfate (50 μg/mL).

### Antibodies, drugs and shRNA reagents

The following antibodies were used: Tsc2, Atg5, Vps34, pS6 ribosomal, S6 ribosomal protein, pAkt, total Akt (Cell Signaling Technology), LC3 (Novus Biologicals), actin (Sigma-Aldrich). Rapamycin and Torin1 were purchased from LC Laboratories. EIPA was purchased from Sigma-Aldrich. SAR405 was purchased from ApexBio (Houston, TX). shRNA targeting Vps34 were obtained from Sigma-Aldrich: TRCN0000322313 (shPI3KC3-1) and TRCN0000350673 (shPI3KC3-2). Pools of cells with stable shRNA expression were selected using puromycin (10ug/ml) and knockdown of Pik3c3 was validated using qRT-PCR and immunoblotting.

### Macropinocytosis uptake assays and flow cytometry

Cells were seeded in 6-well plates and grown in 1% FBS DMEM overnight. Treatments with rapamycin, Torin1, SAR405 and EIPA were carried out for 24 hours followed by addition of 0.5 mg/ml 70 kDa FITC-Dextran (Invitrogen, Carlsbad, CA, USA) for 1 hour as described previously^38^. Cells were subsequently washed twice with ice-cold PBS, trypsinized and recovered in 2% serum phenol red-free DMEM and centrifuged 425 × g for 2 minutes. The pelleted cells were resuspended in 300 ul of serum-free, phenol red-free DMEM and kept on ice. Fluorescence was assessed by flow cytometry (BD FACS Canto II, BD Biosciences), and analyzed with FlowJo analytical software (Treestar). Median fluorescence intensity of FITC (Dextran) or APC (DQ-BSA) were measured in each sample and values were normalized to those of Tsc2-expressing unstained cells.

### Confocal microscopy

Cells were seeded on 4-chamber tissue culture glass slides using 1% FBS DMEM overnight. Cells were treated with inhibitors and FITC-Dextran was added as described in the previous section. Cells were then rinsed with PBS twice and fixed with 4% paraformaldehyde. Images were captured with a FluoView FV-10i Olympus Laser Point Scanning Confocal Microscope using a 60x objective. Confocal filters (Excitation/Emission nm) used for microscopy imaging were: 358⁄461 (DAPI), 494/521 (FITC-Dextran).

### Crystal violet assay

Cells were seeded at 1,000 cells/well density in 96-well plates. After treatment for 24, 48, or 72 hours, cells were fixed with 10% formalin for 10 minutes, stained with 0.05% crystal violet in distilled water for 30 minutes, washed two times, and air-dried. Crystal violet was solubilized with 100 ml of methanol and measured with a plate reader (OD 540; BioTek, Winooski, VT, USA).

### Animal studies

All animal studies were performed in accordance with institutional protocols approved by Boston Children’s Hospital Animal Care and Use Committee. Xenografts were generated by subcutaneously injecting (2.5 × 10^6^) cells mixed with matrigel 1:1 (v/v) (Corning, 356237) to a final volume of 100–150 ul unilaterally into the shoulders of anesthetized, immunocompromised mice using a 21 G needle. Tsc2^−/−^ shCtl and Tsc2^−/−^ shPI3K3C3 tumors were generated in triple immunodeficient NOD-scid IL2Rgamma^null^ mice obtained from Taconic. Mice were inspected weekly and tumors were measured every two days by caliper once they became palpable and >100 mm^3^.

### Statistical analyses

Normally distributed data were analyzed for statistical significance with Student’s unpaired t-test and multiple comparisons were made with one-way and two-way ANOVAs with Bonferroni correction. *In vivo* data are presented as the mean +/− 95% confidence interval (CI) and *in vitro* studies are presented as the mean +/− standard deviation (SD). (GraphPad Prism version 6; GraphPad Software, www.graphpad.com). Statistical significance was defined as p < 0.05.

## Electronic supplementary material

Supplementary Material
