# Supplementary material for: Vps34-mediated macropinocytosis in Tuberous Sclerosis Complex 2-deficient cells supports tumorigenesis
Source: Sci Rep. 2018 Sep 21;8:14161. doi: 10.1038/s41598-018-32256-x (PMC6155086; doi:10.1038/s41598-018-32256-x)
Supplement: Supplementary file 1 — Supplementary Material [file 41598_2018_32256_MOESM1_ESM.pdf]

Vps34-mediated macropinocytosis in Tuberous Sclerosis Complex 2-deficient cells supports tumorigenesis

Authors: Harilaos Filippakis[1]*, Amine Belaid[1], Brian Siroky[2], Constance Wu[1], Nicola Alesi[1], Thomas Hougard[1], Julie Nijmeh[1], Hilaire C. Lam[1], Elizabeth P. Henske[1]*.

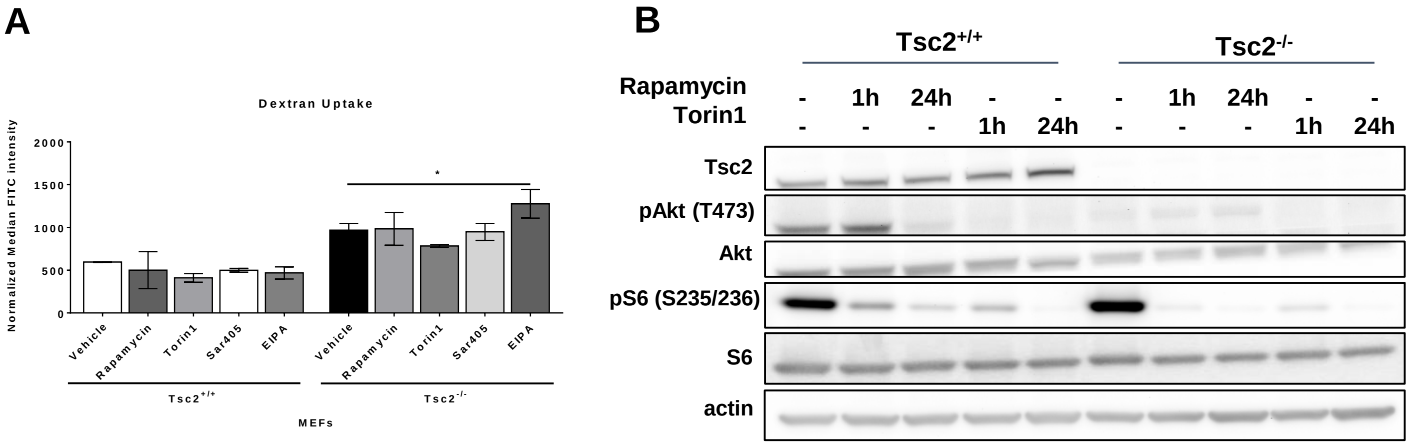

Supplementary Figure 1. (A) Uptake of macropinocytotic cargo dextran (FITC-Dextran, 70 kDa; 0.5mg/ml) was significantly increased in Tsc2$^{-/-}$ MEFs compared to Tsc2$^{+/+}$ MEFs. One-hour treatment with Rapamycin (20nM), Torin1 (250nM), SAR405 (2uM) or EIPA (25uM) had no impact on macropinocytosis in neither Tsc2$^{+/+}$ or Tsc2$^{-/-}$ MEFs. EIPA treatment slightly increased macropinocytosis in Tsc2$^{-/-}$ MEFs. Whole blot shown in supplementary figure 5.

Supplementary Figure 1

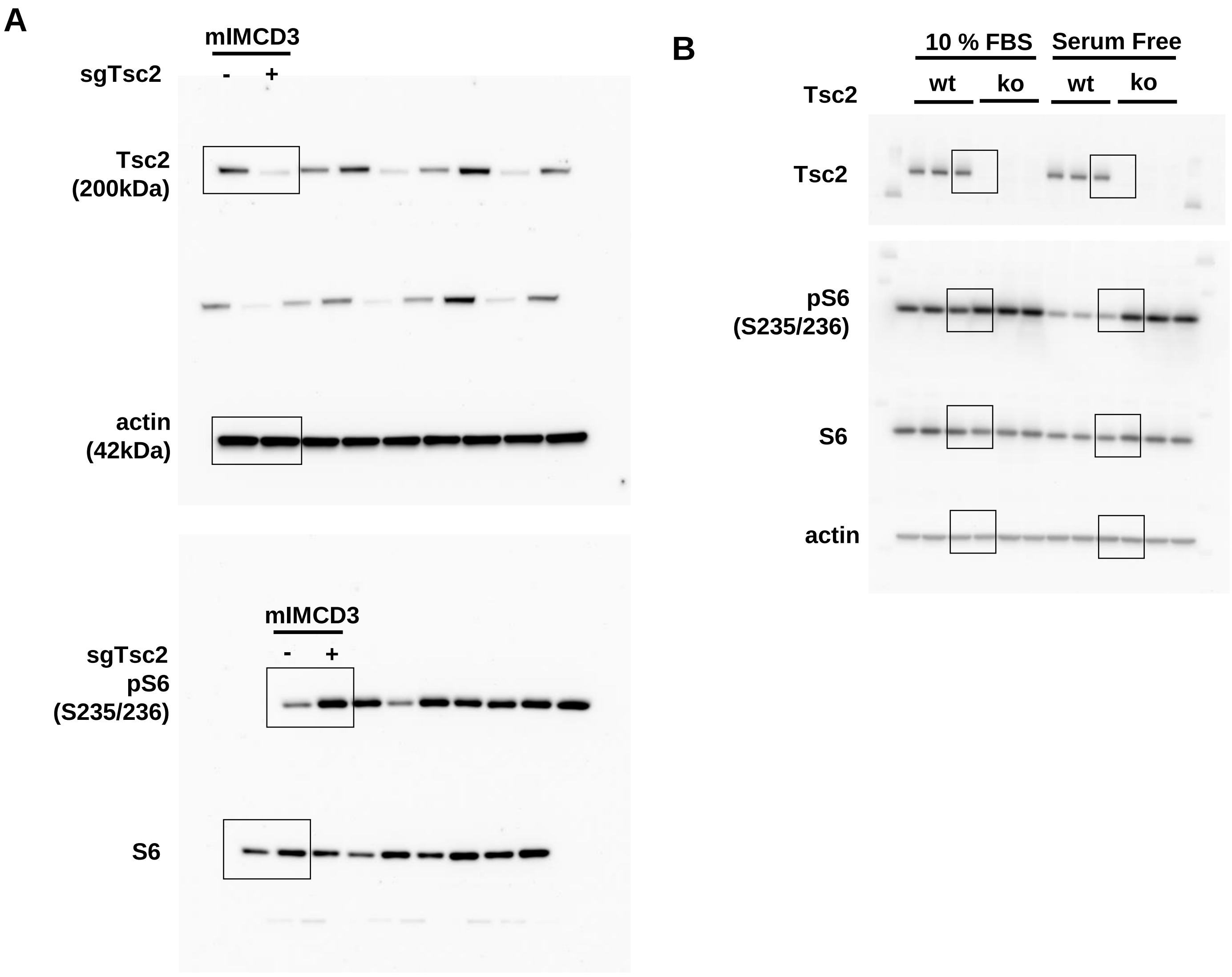

Supplementary Figure 2. (A) Full length immunoblots confirming Tsc2 knockout in mIMCD3 cells. Cropped blot shown in Figure 1C. (B) Full length immunoblots showing mTORC1 signaling in Tsc2wt and Tsc2ko cells grown in 10% FBS or serum free conditions. Cropped blot shown in Figure 1E.

Supplementary Figure 2

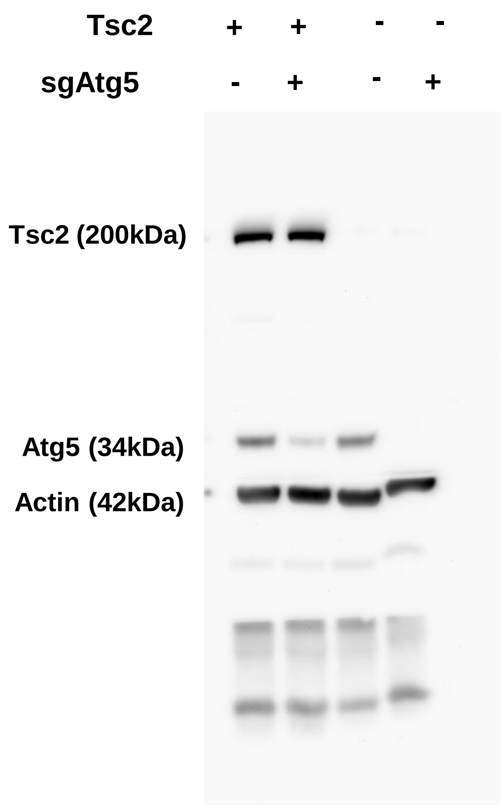

Supplementary Figure 3. Full length immunoblots confirming Atg5 knockout in Tsc2$^{+/+}$ and Tsc2$^{-/-}$ MEFs. Cropped blot shown in Figure 2A.

A

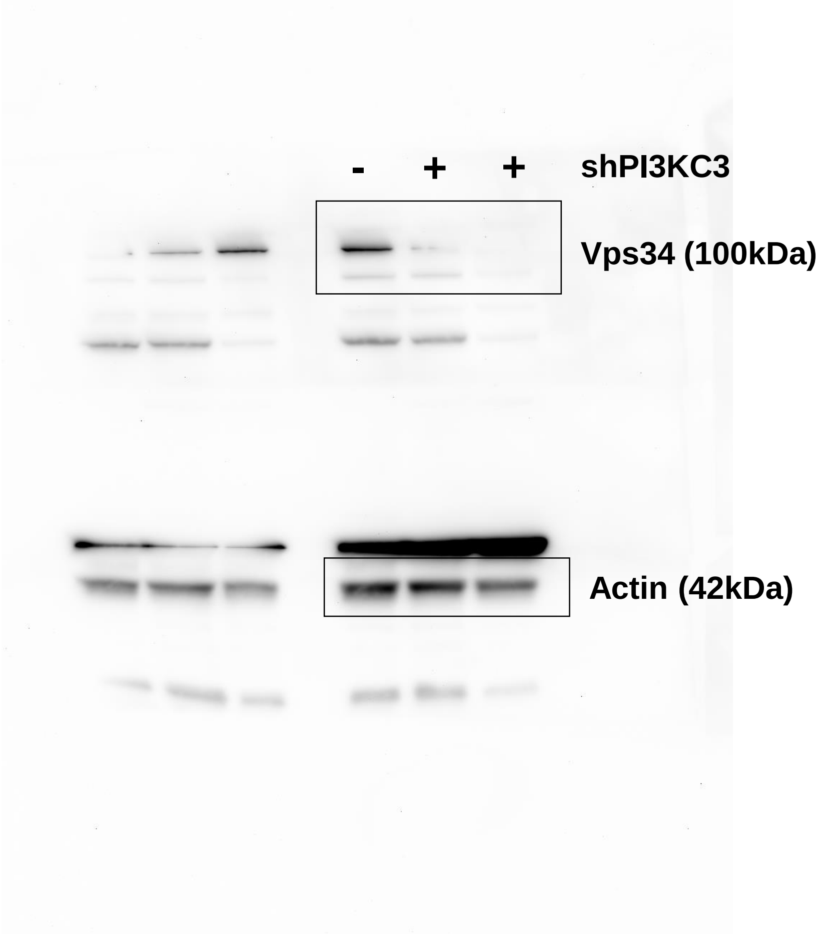

Supplementary Figure 4. Full length immunoblots confirming Vps34 downregulation in Tsc2$^{-/-}$ MEFs. Cropped blot shown in Figure 3C.

Supplementary Figure 4

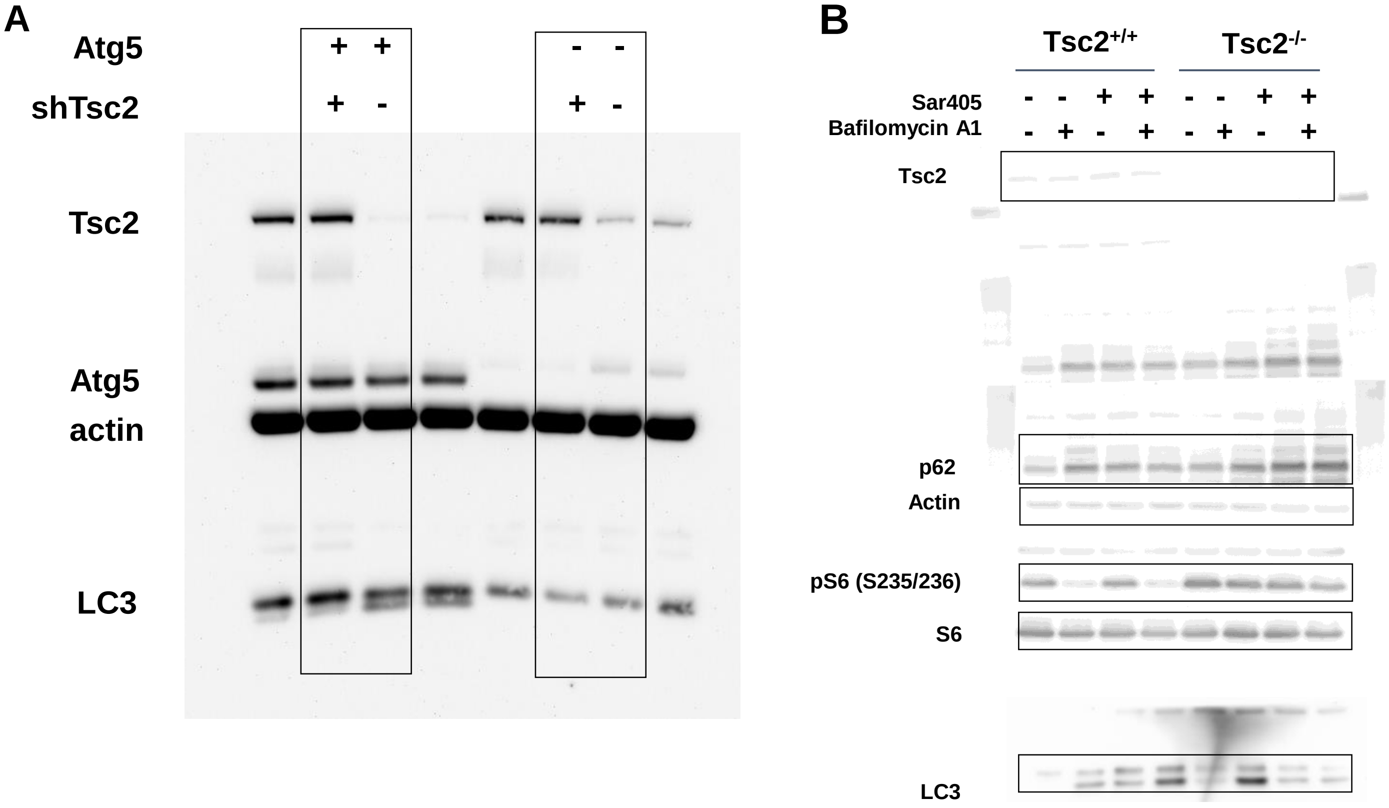

Supplementary Figure 5. (A) Full length immunoblots confirming Tsc2 downregulation in $Atg5^{+/+}$ and $Atg5^{-/-}$ MEFs. Cropped blot shown in Figure 4A. (B) Full length immunoblots showing autophagic flux in $Tsc2^{+/+}$ and $Tsc2^{-/-}$ MEFs following Bafilomycin A1 (50nM), SAR405 (2uM) or combination treatments. Cropped blot shown in Figure 3F.

Supplementary Figure 5

	Macropinocytosis levels	
	$Tsc2^{+/+}$	$Tsc2^{-/-}$
**Basal**	*	***
**Rapamycin (1 hour)**	*	***
**Rapamycin (24 hours)**	*	***
**Torin1 (1 hour)**	*	***
**Torin1 (24 hours)**	*	*
**SAR405 (1 hour)**	*	***
**SAR405 (24 hours)**	*	**
**EIPA (1 hour)**	*	***
**EIPA (24 hours)**	*	*

Supplementary Table 1. Levels of macropinocytosis in $Tsc2^{+/+}$ and $Tsc2^{-/-}$ MEFs following treatments with Rapamycin, Torin1, SAR405 or EIPA. Asterisks denote increasing levels of dextran uptake. *=low levels, **=intermediate levels, ***=elevated levels.
